# The office work and stretch training (OST) study: an individualized and standardized approach for reducing musculoskeletal disorders in office workers

**DOI:** 10.1186/s12995-018-0220-y

**Published:** 2018-12-17

**Authors:** Fabian Holzgreve, Laura Maltry, Jasmin Lampe, Helmut Schmidt, Andreas Bader, Julia Rey, David A. Groneberg, Anke van Mark, Daniela Ohlendorf

**Affiliations:** 1 Institute of Occupational Medicine, Social Medicine and Environmental Medicine, Goethe- University, Theodor-Stern-Kai 7, Building 9a, 60596 Frankfurt am Main, Germany; 2 Managing Director, Health and Safety, Daimler AG, Stuttgart, Germany; 3 Manager Corporate Health Promotion, Health and Safety, Daimler AG, Stuttgart, Germany; 4 Institute of Biostatistics and Mathematical Modeling, Goethe-University, Frankfurt/Main, Germany

## Abstract

**Background:**

Musculoskeletal disorders (MSD) are a common health problem in office workers. In Germany, MSD (mainly back pain related) are the main cause of workdays lost to incapacity. This is not only bothersome for the employees, but also causes higher costs for the health system and employers. Workplace health promotion programmes (WHPP) can help to reduce this as they reach large target groups and are easily accessible. In this context, stretch training exercises have already proven to be effective. In the present study, a new approach focusing on trunk extension is to be investigated.

**Methods:**

To evaluate the training device “five-Business”, 250 office workers will train two times a week for 3 months. The control group will consist of 100 office employees. The device “five-Business” enables five different full body exercises. The intervention will be evaluated before week one and after week twelve via three assessments: a) the Short Form-36 (SF-36) to record the general health status and health-related quality of life, taking into account physical, psychological and social factors, b) the Nordic Questionnaire to evaluate complaints of the musculoskeletal system, c) Range of Motion (ROM) measurements using a digital inclinometer and a measuring tape respectively.

**Conclusion:**

The “five-Business” combines elements of yoga and the McKenzie fundamentals, taking into account the Myers myofascial pathways in a highly torso-oriented, standardized stretching program. Due to the given exercise execution on the device and the individual adjustment possibilities of the stretching position (body size and range of motion) by the abutment, all exercises are individualized and standardized at the same time. In comparison to existing stretching interventions, this is a new approach in the framework of reducing musculoskeletal disorders and improving the quality of life in workplace health promotion.

## Background

In industrial nations, the service sector is the dominant economic sector [1, 2], where the majority of working time is spent in a sitting position [3]. About 50% of the employees suffer from moderate pain and about 30% from severe back and neck pain [4, 5]. Risk factors include years spent in an office [6], gender [5–7], body mass index (BMI) [7] and age [5–8]. The weighting of the influencing factors can vary depending on the cultural context, as observed in a comparison between Malaysian and Australian office workers [9]. If diseases of the musculoskeletal system (MSD) are not treated, this can ultimately lead to a greater number of work days lost to incapacity [6, 10–12]. One reason might be, that in physical therapy of MSD occupational factors are not consistently taken into account [13]. In Germany, MSD are the main cause of disability days [14, 15]. From 2011 to 2017, MSD-related diagnoses accounted for an average of 22% of all diagnoses [14]. These were mainly back pain related [15]. In the worst cases, these complaints can become chronic and the temporary inability to work can become an occupational disability. The high rate of MSD related absenteeism [14] is not only a burden for the employees, but also causes greater costs for employers and the health system. In 2016, the loss of gross value added caused by MSD in Germany amounted to 30.4 billion euros, which is equivalent to 1% of gross national income [16].

In the past, behavior oriented prevention and structural prevention in the form of WHPP appears to make sense, as they reach large target groups and are easily accessible [8, 17–19]. Usually these programmes pursue one of the following strategies: workplace optimisation, workplace policy changes (structural workflow, for example, standing meetings), provision of information (to improve lifestyle and physical activity levels) or multi-modal interventions [17, 20]. Interventions of this kind have been studied intensively. However, meta-analyses found only minor positive effects on physical activity [2, 8], reduction of sitting time [20], weight and lifestyle [8]. The study designs of the included studies were very inconsistent with regard to sample size (*n* = 40–924), intervention period (9 weeks to 2 years) [8], grouping and target sizes. In total, younger volunteers (≤40 years) seemed to benefit more in these studies [8].

**Fig. 1:**
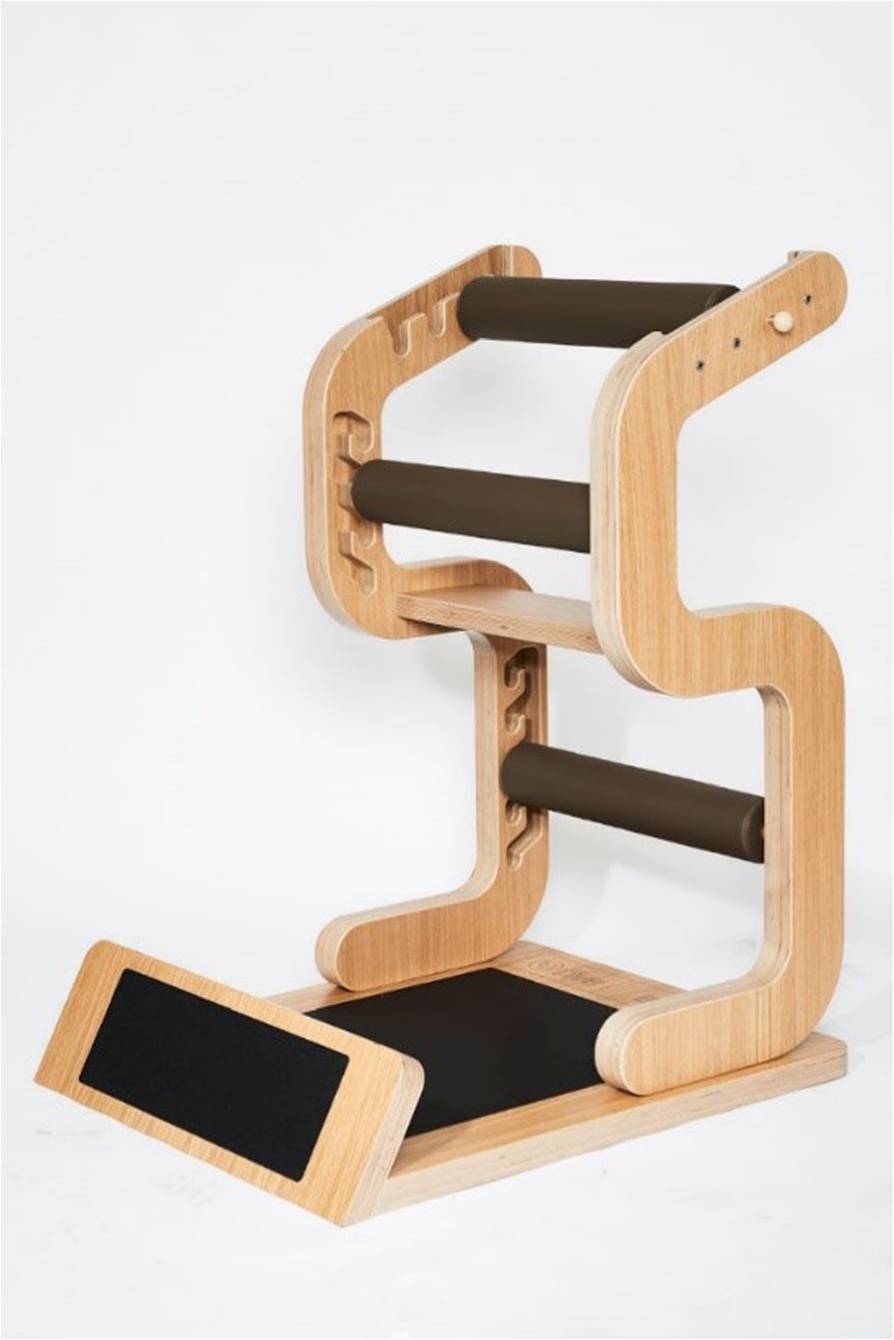
Training Device “five-Business”: The black rolls are adjustable to individual sice, flexibility and skill of the user

Although they are far less extensively studied, instructed stretching programmes at the workplace offer a more promising approach [12, 21–24]. For example, the research of Tunwattanapong et al. [21] demonstrated a reduction in neck and shoulder complaints (− 1.4; 95% CI: -2.2, − 0.7 using the visual analogue scale) and an improvement in quality of life (14.0; 95% CI: 7.1, 20.9 using the physical dimension of SF-36). However, to our knowledge, the investigated programs were neither standardized nor individualized, which could be important if a heterogeneous population, such as the many employees present in an office building, is to be reached. Therefore, a new and more global approach, the “five-Business” stretch training programme (“Five-Konzept”, Hüfingen, Germany), is presented in this study. Here, the exercises are executed on an adjustable wooden device (Fig. 1).

The objectives of the present study were to evaluate the “five-Business” stretch training programme in terms of its effects on ROM, its effectiveness in reducing MSD and its effects on the health-related quality of life.

### “Five-Konzept”

Basically, the training method of “Five-Konzept” is a static and predominantly passive set of stretching exercises which is carried out on a special wooden device. Taking into account the course of the so-called myofascial pathways according to Myers [25], whole muscle chains are intensively stretched with isometric contraction. The focus of the exercise programme is on the musculature of the trunk, especially the extension of the spine, where the exercises partly resemble yoga positions.

Since sitting usually involves flexion of the spine, these exercises can be seen as a counter-movement to sitting. This variable compression of the spinal discs improves their nutrition through diffusion [26]. For this reason, especially people with a sedentary lifestyle, such as office workers, can benefit from this programme.

Currently, this training method is mainly used by fitness providers throughout Germany, although no scientific studies of this new training concept have been published to date. However, it has already been shown that workplace-specific yoga training (Dru-Yoga) can contribute to the reduction of back pain and stress, as well as to the improvement of well-being (PANAS-X questionnaire) [27, 28]. The “five-Business” also includes the treatment in the trunk extension often recommended by McKenzie’s treatment concept [29, 30].

A special device (“five-Business” device, see Fig. 1) was developed for the application of the “Five-Konzept” in the context of WHPP. The device allows an individual setting for all exercises and summarizes the “Five-Konzept” validly.

Although the method is named “active muscle length training” rather than “stretching” by the provider, the authors decided to use the term stretching as, from a physiological point of view, this is the correct term.

### Aims

Chronic disorders, as a result of persistent complaints in the sense of upper and low back pain, are often associated with mood disorders and a poorer quality of life [31].

This pilot study will evaluate the effectiveness of systematic stretch training exercises of the trunk in office workers at the workplace. The influence of the training on the quality of life, the muscular skeletal discomfort and the mobility of the stretched structures will be investigated. On the basis of these results, it can then be evaluated to what extent the training is suitable as a WHPP measure.

### Hypotheses

#### Hypothesis 1

A systematic, occupation-specific and guided stretch training programme (“Five Konzept”) leads to an improvement of the quality of life.

#### Hypothesis 2

A systematic, occupation-specific and guided stretch training programme (“Five Konzept”) leads to a reduction of MSD, especially in the lower back area.

#### Hypothesis 3

A systematic, occupation-specific and guided stretch training programme (“Five Konzept”) leads to an increased mobility of the stretched structures.

## Materials and methods

### Subjects

Within the framework of the intervention control study, a total of 350 subjects aged between 18 and 65 years are to be measured, 250 of whom belong to the intervention group and a further 100 to the control group, the allocation being nonprobabilistic. All subjects work full-time at office workplaces. Exclusion criteria are subjects who have had relevant operations or who have surgical stiffening of the musculoskeletal system, relevant artificial joint replacement, severe diseases such as ankylosing spondylitis, chronic destructive joint diseases, multiple sclerosis, myodystrophic or neurodegenerative diseases, congenital malpositions of the musculoskeletal system or an acute herniated disc. In addition, the intake of muscle relaxants or other drugs that influence the elasticity of the musculature and pregnancy are considered contra indicators.

All participants will provide written informed consent to take part in the study in advance. The study is approved by the ethics committee of the Medical Faculty of the Landesärztekammer Baden-Württemberg (F-2017-073).

### Recruitment

The program will be promoted via in-house e-mails from the health department of the respective company and, at the same time, the possibility of registering for the study will be offered. The registration period is set for 2 weeks. Afterwards, all potential participants will be contacted by telephone to clarify the exclusion criteria and to divide them into intervention groups based on availability.

### Intervention program

The intervention program “five-Business” has been designed by the commercial provider “Five-Konzept” (Hüfingen/Germany) for the implementation in company settings and for health promotion. All exercises are performed standing on one machine, wearing shoes (for safety reasons subjects were not allowed to wear heeled shoes > 5 cm) and in “working clothes” (Fig. 1). The dimensions of the device are 116 cm × 82 cm × 128 cm and the weight is 60 kg.

The subjects perform the following 5 exercises: (1) Stand, (2) Chest, (3) Ischio, (4) Hip and (5) Lateral.

**Fig. 2:**
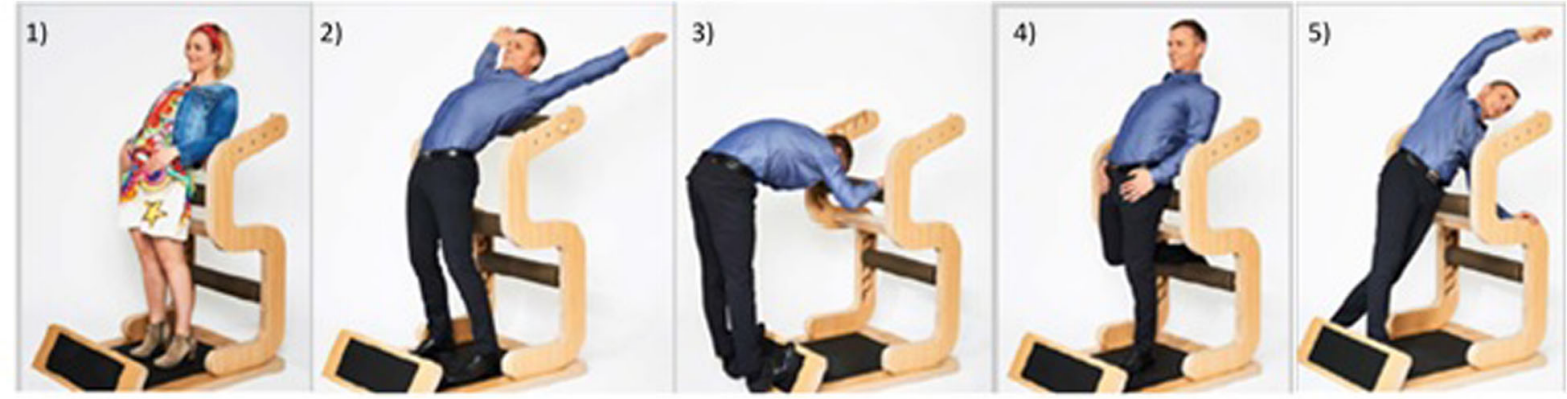
All 5 exercises of the “five-Business”: exercises in the order of execution: 1) Stand, 2) Chest, 3) Ischio, 4) Hip and 5) Lateral

1. Stand: In the exercise “stand”, knee, hip, lumbar spine and thoracic spine are extended to the maximum (Fig.2).
  The cervical spine, on the other hand, is flexed. Following the myofascial meridians after Myers [25], structures of the superficial frontal line (SFL) ((sternalis muscle), sternochondral fascia, rectus abdominis muscle, quadriceps femoris muscle in particular rectus femoris muscle and patellar tendon) are statically passively stretched. For the sake of completeness, a selection of structures of the deep frontal line (DFL), which are potentially strained due to the direction of movement, are listed below (endothoracic fascia, transversusthoracis muscle, pericardium, mediastinum, parietal pleura, diaphragm, anterior longitudinal ligament, sacral fascia, psoas muscle, iliac muscle, pectineus muscle, femoral triangle, medial intermuscular septum of the thigh, adductor brevis muscle and adductor longus muscle). By increasing the torque (e.g. by raising the arms), both the strain stress and the isometric contraction of the stretched musculature can be increased.
2. Chest: A back bend is carried out analogous to “Stand” and “Hip”. The abutment is positioned at the level of the shoulder blades; this brings the strain stress especially to the chest area. Structures of the deep frontal armline (DFA) [25] (minor pectoralis muscle, clavipectoral fascia, biceps brachii muscle, anterior edge of the radial periosteum, muscles of the ball of the thumb and radial collateral carpia), of the superficial frontal armline (SFA) [25] (pectoralis major muscle, latissimus dorsi muscle, septum intermusculare brachii mediale, flexors and carpal tunnel), the functional frontal line (FFL) [25] (pectoralis major muscle, rectus abdominis muscle and adductor longus muscle), as well as the upper SFL and DFL, are stretched. By actively returning the arms, the stretching stress in the chest area is increased so that the exercise can mainly be assigned to active static stretching.
3. Ischio: Static passive stretching of the entire superficial back line (SBL) [25] (galea aponeurotica, epicranial fascia, sacrolumbar fascia, erector spinae muscle, sacrotuberous ligament, ischiocrural musculature, gastrocnemius muscle, achilles tendon, plantar fascia and short toe flexors) is performed by extension in the ankle and knee joints, as well as flexion in the hip and spinal column.
4. Hip: In principle, the same structures are used as for “Stand” (SFL/DFL). A special feature is the arbitrary isometric contraction of the agonists. This is ensured by strong pressing of the hung-up foot. The focus of the “Hip” exercise is on the strain on the hip-bending muscles and the associated structures.
5. Lateral: During lateral flexion, passive static stretching is performed under consideration of the lateral line (LL) [25] (splenius capitis muscle, sternocleidomastoid muscle, intercostal internal/external muscle, obliquus internal/external muscle, gluteus maximus muscle, gluteus medius muscle, tensor fascia latae muscle, iliotibial tract, anterior ligament of the head of the fibula and the peroneus longus/previs muscles). The torque can be increased by elevating both arms or holding weights above the head. This results in both increased stretching stress and increased isometric contraction of the stretched musculature.

### Questionnaires

#### Nordic questionnaire

The Nordic Questionnaire records complaints of the musculoskeletal system and was developed by Kuorinka et al. in 1987 [32]. It has been validated and is used internationally in a wide variety of occupational groups such as administrative occupations [33–36], factory workers [37–40] or health professions [41–44]. Basically the Nordic Questionnaire asks general information, questions about the person, habits and work situation, but also provides overviews - divided into body regions - of the 7-day and 12-month prevalence of complaints, as well as the lifetime prevalence of complaints and functional impairments to date. Finally, it provides information on the focal points [1] neck, [2] shoulder and [3] lumbar spine in terms of the duration and frequency of complaints, impairment of work and leisure activities, as well as doctor consultation and the inability to work. This questionnaire is aimed at chronic and acute complaints of the musculoskeletal system, consisting of a 7-page survey which can be completed by ticking the appropriate box and which takes 15 to 20 min to complete.

#### Sf-36

The Short-Form-36 questionnaire (SF-36), developed by Ware and Sherbourne in 1992 in the United States [45], measures the general health status and health-related quality of life, taking into account physical, psychological and social factors. Currently, the SF-36 is widely used internationally [46–50]. The test residual reliability of the German version of the SF-36 varies over the individual subscales between *r* = .67 and *r* = .85. In a study with back pain patients (*n* = 243), the internal consistencies for all subscales were determined (α = .60–.93) [51]. For the change sensitivity in lumbar back pain, low to moderate effect sizes are reported for the individual subscales (Standardized Effect Size (SES): (1).48; (2).13; (3) .20; (4) .39; (5).75; (6).28; (7).58; (8).21; (9)-.09; (10).32) [52]. The questionnaire responses are recorded as individual items and as sum scores, listed in a data table which serves as a basis for further statistical analysis.

### Range of motion measurements

Range of Motion measurements are used to evaluate the effectiveness of the individual exercises with regard to changes in the stressed active and passive structures. The selection of the tests to be used are congruent with the stretched muscle chains of the individual exercises (Fig. 1). Two different measuring methods are used to measure the degree of mobility: a measuring tape (exercises 3 & 5) [53–58] and a digital inclinometer (exercises 1, 2 & 4) [59–69]. The digital inclinometer (Model: Acumar™ DIGITAL INCLINOMETER Model ACU002 / Lafayette Instrument Company / Lafayette / USA) is equivalent to the goniometer in terms of measurement accuracy [60], but is also superior in some validation studies (interrater reliability (*r* = .92 to *r* = .53 [66], intrarater reliability [60, 67]. Antonaci and colleagues [59] recommend the use of a digital inclinometer in clinical investigations. Reliability studies for shoulder mobility report an intrarater correlation between ICC = .65 and ICC = .96 [62, 64, 68]. Intrarater correlations between *r* = .89 and *r* = .94 were found for cervical spine mobility [63]. For interrater reliability, correlations between ICC = .58 and ICC = .95 for the shoulder [62, 64, 65] and between *r* = .81 and *r* = .84 [63] for the cervical spine were given. For the responsivity of the shoulder, Valentine and Lewis [68] were able to determine measurement errors of 1.3° for external rotation, 2.3° for internal rotation, 4.8° for abduction and 3.9° for flexion, thus, changes from 5° - 10° can be measured. Furthermore, Kolber et al. [65] determined the smallest detectable interrater difference (MDC (90)) to be 8° (flexion), 4° (abduction), 9° (external rotation) and 8° (internal rotation).

The sports engine tests are listed according to the sequence of measurements to be carried out.

### Shoulder test modified after Janda

This test is intended to show changes caused by the exercise “Chest”. In order to determine the mobility of the shoulder joint, especially of the pectoralis major muscle, the Janda examination is performed in a modified form [70]. In contrast to Janda, the elbow is stretched and the inclinometer is placed proximal to the processus styloideus radii on the radius. The measurement of the middle and upper sternal part of the pectoralis major muscle is carried out at approximately 90° abduction and rotated outside.

### Modified Thomas test

The modified Thomas test is used to check for changes in flexibility in the hip-bending musculature. High interrater reliability ranges are given for the use of an inclinometer and goniometer have been determined (*r* = .91–.93; ICC = .89–.92). The intrarater parallel-forms reliability for the measurements made by the same examiner with both measuring instruments is *r* = .89–.92; ICC = .91–.93 [69]. In order to obtain valid results, the pelvic inclination must be controlled [71]. The pelvic inclination was standardized by placing the digital inclinometer from the anterior superior iliac spine downwards. In this position, the alignment of the pelvis is set to 0°. The inclinometer is then placed on the thigh, above the patella, to determine the joint angle.

### Retroflexion of the trunk after Janda in the modified version

The retroflexion of the lumbar spine and thoracic spine, in particular, is checked by means of the modified retroflexion test according to Janda [70]. Since both ends of this range of motion are difficult to fix, angle measurement is only possible with difficulty. In order to counteract pelvic rotation in the sagittal plane, the pelvis was fixed to the treatment couch at the level of the posterior superior iliac spina with a tensioning strap. Furthermore, unlike Janda, the angle of the elbow is not used as a parameter for torso extension, but the position of the thoracic spine in the sagittal plane is determined by placing the inclinometer on the sternum.

### Fingertip-to-floor test

The “fingertip-to-floor” test is used to evaluate the “Ischio” exercise. The aim is to assess the mobility of the back, both hips, the ischiocrural musculature and the neuromeningeal structures. The changes are measured using a conventional measuring tape. The reliability lies between *r* = .76 and *r* = .99 [53, 54, 56, 72] and shows a good sensitivity for changes [55].

### Lateral inclination

The test of the lateral inclination evaluates the “Lateral” exercise. This is measured by the maximum lateral inclination with a standardized stand position. Sagittal fluctuations in the lateral inclination are eliminated by leaning the back against a wall. The lateral finger-to-ground distance is measured using a measuring tape [66].

### Measurement protocol

The intervention study is scheduled to last 12 weeks. In the week before and the week after the study, the Range of Motion measurements and the surveys (Nordic Questionnaire and SF-36) will be carried out. For reasons of practicability, a randomization of the measurement sequence is deliberately omitted. The test persons complete the stretch training twice a week for approximately 10 min. Each exercise is held twice for 20 s; the time period is measured with a timer. The training is accompanied and controlled by trained trainer personnel throughout the intervention and only one-to-one supervision takes place. Where possible, attention is also paid to progressive load control; for this purpose, the training device is marked in advance so that the device setting and the stand position can be registered over the course of the training.

In order to be able to trace actual changes back to the intervention, the test persons must keep a training diary in order to identify influences from private physical activities. Since, over a total period of 14 weeks, including days for holidays, illness and business trips, it is not possible for office workers to participate consistently in training both during the study and in real working life. Therefore, two goodwill appointments are offered so that at the end of the intervention period the test persons must have completed 22–24 training appointments. In the control group, the Range of Motion measurements and surveys (Nordic Questionnaire and SF-36) are carried out at 12-week intervals, analogously to the intervention group.

In order to achieve the most accurate measurement results possible, the measurements should always be carried out by the same experienced investigator [62, 73]. All measurements are performed three times, from which the mean value is calculated for further statistical analysis [74].

### Evaluation criteria

The units of measurement are centimetres and degrees, as well as sum scores and information from the questionnaires according to the respective question.

### Statistical data analysis

The statistics program “IBM SPSS Statistics 25” is used for the statistical evaluation. Firstly, all collected data are tested for normal distribution with the Kolmogoroff-Smirnoff-Lilliefors-Test.

For normally distributed data, the T-test is used for paired samples, whilst for non-normally distributed data, the Wilcoxon matched pairs test is used (hypotheses 1 & 3). Since the results of the Nordic Questionnaires are nominal or ordinal, the Chi-square test is used to test hypothesis 2 for independent group comparisons. In the pre-post comparison, the McNemar test for paired samples, or the Cochrans-Q test for repeated measurements, is performed.

The independent T-test (normally distributed) and the Wilcoxon-Mann-Whitney-U-test (non-normally distributed, metric and ordinal) are used to compare the values between the intervention and control groups for metric values. The Chi-square test is used to check nominally scaled values.

Furthermore, correlations between complaints, changes in range of motion and quality of life, are to be tested. For this purpose, the Pearson correlation is performed for normally distributed values and the Spearman correlation for non-normally distributed values.

The statistical evaluation is carried out under the supervision and advice of Dr. J. Rey (Institute for Biostatistics and Mathematical Modelling of the Medical Department of the Goethe University Frankfurt).

## Discussion

The combination of elements of yoga and the McKenzie fundamentals, taking into account the Myers myofascial pathways in a highly torso-oriented, standardized stretching program, could provide new approaches to reducing MSD and improving the quality of life in workplace health promotion [25, 27–30]. Device-supported mobility training is suitable for integration into everyday office life as it can be carried out quickly and easily on a single device (TÜV tested). Due to the given exercise execution on the device and the individual adjustment possibilities of the stretching position (body size and range of motion) by the abutment, all exercises are individualized and standardized at the same time. Thus, it can also be used for heterogeneous groups in terms of physical proportions and capabilities. This is also an advantage over the stretching interventions evaluated so far [12, 21–24]. Shariat et al. [12, 23] evaluated 13 stretching exercises for the neck, shoulders and trunk, which were demonstrated in a video clip. After a two-week familiarization phase, the subjects trained three times a week with progressive exercise duration. A supervisor was available for questions and occasional monitoring. After eleven weeks, a reduction in pain in the trained areas, greater mobility and less perceived exertion could be observed. Using similar stretching exercises, Tunwattanapong et al. [21] also found positive effects in reducing neck pain and improving quality of life. However, the intervention group also received parallel instructions on ergonomic sitting. It is, therefore, unclear as to what influence the stretching had itself. However, the improvements observed were greater in subjects who had stretched at least three times a week than in those who had trained less frequently. In both interventions, the exercises were neither individualized nor standardized. In addition, a broad approach was chosen in both cases, taking into account not only the trunk but also the shoulders/arms and neck. The approach of a short, mainly torso-oriented stretching training exercise using myofascial muscle chains has not yet been investigated.

This stretch training exercise, developed for recreational sports, is now to be used within the framework of a WHPP to stretch muscle chains as a whole (especially in the trunk and hip area), which remain in a flexed position for several hours a day by sitting for long periods in a rigid, unfavorable manner. Despite the positive evidence described for the efficacy of similar stretching exercises, scientific studies have not found any connection between sitting per sé and low back pain [75] so far. Whether this also applies to other forms of MSD has not yet been clarified. Nevertheless, a concrete comparison of the known stretching techniques and the “five Business”, with regard to the influence on the quality of life and prevalence of MSD, should be sought in the future.

A main problem concerning the implementation of WHPP is the small number of participants in relation to the workforce addressed (2–60%) [8] and, also the long-term motivation of the employees. A device installed on site, that promotes an intuitive execution of the exercise and functions as a constantly present “reminder” of the training itself, could promote compliance. In further studies, it should be examined whether the “five-Business” is superior to conventional stretching in this respect. This could also depend on the individuals psychosocial workplace risk, which can be assessed using the Short Questionnaire for Workplace Analysis (KFZA) [76]. Overall, as the physical constitution of office workers cannot be changed and the postural demands can not be significantly altered, it is advisable to optimize health prevention, such as has already been the case with exposure factors in physiotherapists [77] and musicians [78].

### Limitations

An evaluation of the intervention, by means of the Range of Motion measurements, only records a change in mobility in the form of an altered movement amplitude. Through the application of the intervention, not only improvements in extensibility but also changes in strength, especially in isometric strength, can be expected. An effective measurement of the isometric maximum force is not planned for reasons of methodical implementation (material, temporal and spatial resources). Since the exercises Stand, Chest and Hip (with the exception of slightly different focal points) all include the back bend, no clear tests can be assigned to these exercises. Instead, in order to determine in which structure any changes occur, the hip and spine mobility in the extension direction is tested separately. Therefore, the modified Thomas test is used to examine the hip-bending musculature, while retroflexion of the trunk increasingly focuses on the mobility of the lumbar and thoracic spine. In addition to the modified shoulder test according to Janda, it should be noted that the determined joint angle includes the mobility of the elbow. This is particularly important in cases of muscular limitation of the elbow extension.

It should be noted that the muscle chains postulated by Myers have not yet been sufficiently investigated. Although there is strong evidence for SBL and FFL and moderate to strong evidence for LL, there is still no evidence for the existence of SFL [79].

When measuring mobility, the time of day plays a decisive role. Accordingly, the fingertip-to-floor distance decreases significantly in the course of the day [80]. A standardization of the time of day will only be possible approximately, in view of the availability of the office employees. Furthermore, an optimally standardized regular participation over a period of 3 months is not possible within the framework of such a study, since days lost due to the incapacity to work or to business or private travel cannot be avoided. Accordingly, the internal validity is not optimal. However, it should be noted at this point that no higher internal validity can be expected if the programme is implemented in the daily work of office employees. In this context motivation and experience of pain is unlikely to be homogenous in all subjects.

In the standardized Nordic Questionnaire, the 12-month pain prevalence is a fixed component. At this point, the query of the 3-month pain prevalence according to the intervention period would increase the change sensitivity. However, a modification of the questionnaire would entail a new evaluation on the one hand, whilst, on the other hand, this would make a comparison with other study data more difficult.

## Conclusion

This projects aims to provide health management departments information on whether a standardized and individualized stretch training exercise has an impact on MSD of the staff. Effective programs are necessary to reduce the high number of work days lost due to incapacity with MSD. Apart from the self reported MSD and quality of life, it is a further goal of this study to investigate if the training affects physiologically measurable ranges of motion. Based on these outcomes, health managers will obtain evidence-based information on which they can decide whether the program is suitable for their company.

## Abbreviations

BMI: Body Mass Index
DFA: Deep Frontal Armline
DFL: Deep Frontal Line
FFL: Functional Frontal Line
LL: Lateral Line
MDC: Smallest detectable interrater difference
MSD: Musculoskeletal Disorders
ROM: Range of Motion
SBL: Superficial Back Line
SES: Standardized Effect Size
SF-36: Short Form 36
SFA: Superficial Frontal Armline
SFL: Superficial Front Line
WHPP: Workplace Health Promotion Programs
